# Influence of Inter-Particle Distances on the Rheological Properties of Cementitious Suspensions

**DOI:** 10.3390/ma14247869

**Published:** 2021-12-19

**Authors:** Rajagopalan Sam Rajadurai, Su-Tae Kang

**Affiliations:** Department of Civil Engineering, Daegu University, Gyeongsan 38453, Korea; samraj9593@gmail.com

**Keywords:** supplementary cementitious materials (SCMs), cementitious suspensions, rheology, inter-particle distances, yield stress

## Abstract

Supplementary cementitious materials (SCMs), such as fly ash (FA), blast furnace slag (BS), and silica fume (SF), have been mostly used as a replacement for Portland cement (PC). Replacing the SCMs with cement can provide improved strength characteristics; however, their applicability depends on the flow characteristics of the fresh mixtures. In this study, the rheological performance of cementitious suspensions in paste scale with different water-to-solid (W/S) volume ratios, varied from 1.25, 1.50, 1.75, 2.00, 2.25, to 2.50, was evaluated. As a result of the rheological tests, the yield stress and plastic viscosity of PC, FA, BS, and SF suspensions decreased as the W/S ratio increased. This study also estimated the inter-particle distances of the cementitious suspensions, and their relationship to the rheological properties was established. The inter-particle distances of the PC, FA, BS, and SF suspensions were in the ranges of 5.74~14.67 µm, 5.18~11.66 µm, 3.82~9.34 µm, and 0.107~0.27 µm, respectively. For very fine particles with a large surface area, the sensitivity to the rheological properties was high and the sensitivity was low when the particle sizes increased, indicating that the rheological properties were more sensitive to fine particles.

## 1. Introduction

Cementitious materials are the most reliable and essential components used for a wide range of construction applications, from buildings to special structures [1,2]. Cementitious materials are mainly classified into the following two types: hydraulic cement and supplementary cementitious materials (SCMs) [3,4,5,6,7]. Portland cement is the most common hydraulic cement that hardens in chemical reactions with water and binds aggregate particles [3,4,5,6,7]. SCMs such as metakaolin, fly ash, blast furnace slag, and silica fume were used with Portland cement to improve its workability, strength, and durability [8,9,10,11,12]. In addition, using SCMs as a partial replacement for cement has a great environmental advantage and contributes significant progress towards sustainable construction [13,14,15,16]. The replacement of Portland cement with SCMs can provide improved strength characteristics and environmental advantages, and the applicability of SCMs depends on the flowability of the fresh mixtures. Further investigation is needed to identify the advantages and limitations of using binary and tertiary fractions. Various studies have previously been designed to investigate the rheological behavior of cement pastes and the effectiveness of using SCMs [17,18,19]. The use of fly ash with cement reduced the water content and prevented the agglomeration of particles due to the very fine particles adsorbed onto the oppositely charged surfaces of the cement particles [20]. The spherical shape and smooth surface of fly ash reduces the inter-particle friction and plastic viscosity [21]. In addition, fly ash particles, mainly composed of the glassy phase, provide a more fluid mixture and reduce the use of water-reducing agents [22,23,24]. The concrete mixtures with fly ash increase the fine fraction of the composition that provides better flowability and a larger paste volume [11]. Replacing the Portland cement with ground-granulated blast furnace slag improves the flowability [25], associated with the smooth surface and less chemical activity [8,9,25]. The granulated blast furnace slag and particle shape influence on the rheological properties was investigated and showed improved fluidity [8,9,25]. Additionally, the surface area and chemical activity of the slag have a great influence on the rheological properties of concrete mixtures [25]. Moreover, adding blast furnace slag to more than 15% of the cement reduces the yield stress and plastic viscosity more efficiently [26,27]. As for the silica fume, it was effective in improving the mechanical properties [28], and the physical effect of particle packing by silica fume reduces the void space [29]. Park et al. found that the yield stress and plastic viscosity increased as the silica fume replacement increased [27]. When silica fume was replaced by 10 and 25% of cement, the mixtures showed low shear-thinning behavior [30]. Salem et al. investigated the rheology with high silica fume replacement rates of 10, 20, 30, and 50%, and found increasing hysteresis in up/down flow curves [31]. Hence, the partial substitution of cement by SCMs can change the rheological properties and stability of the mixtures, which can lead to a greater or lesser packing density of the fine powders and can reduce or increase the inter-particle friction.

The main parameters that influenced the rheological properties of fresh cementitious suspensions were particle size, surface area, packing density, solid volume concentration, and water content, which are also related to the inter-particle distances [32]. In addition, the excess water fills up the voids between the particles, which contributes to the flowability of the fresh cementitious suspensions [33,34]. The large amount of excess water improves separation and dispersion [35], which is present in the form of a water film coating the surface of the particles [36]. The higher amount of excess water forms thicker water films, reducing the friction and interaction between the particles, and eventually leading to higher flowability [37,38]. However, excessive amounts of water can adversely affect the strength and hardened properties of the fresh cementitious suspensions. Therefore, it would be better to increase the flowability of the fresh suspensions by reducing the space between the particles, by increasing the packing density of the particles. The smaller water film thickness (inter-particle spacing) was beneficial to the clustering and bridging of the hydration products, and the densification of microstructures that contributed to the strength development [39,40]. In addition to the flowability of fresh cement paste, the mechanical properties and volume stability of cement paste during hardening depend on the inter-particle spacing [32]. Therefore, there was a relationship between the inter-particle spacing and rheological properties of the cement paste [32]. There are various studies that estimated the inter-particle spacing, and its relationship with the rheological properties was established [41,42]. Kwan et al. developed a wet packing method to measure the packing density of cement pastes and applied the method to eventually determine the water film thickness or inter-particle distances [41,42]. To estimate the inter-particle spaces of the concrete mixtures mixed with SCMs, the first step was to fully understand the properties of the SCMs paste scale, followed by those of the cementitious paste scale (cement + SCMs), mortar scale, and concrete scale. Most of the previous studies focused on estimating the inter-particle spaces of fresh cement paste, mortar, and concrete mixtures. On the other hand, few studies were designed to estimate the inter-particle spaces of supplementary cementitious suspensions.

Therefore, this study was designed to understand the rheological properties of cementitious materials in paste scales, with different W/S ratios ranging from 1.25, 1.50, 1.75, 2.00, 2.25, to 2.50, respectively. The SEM micrographs, chemical compositions, and particle size distribution of the cementitious materials were measured. The rheological properties were evaluated using the Bingham model, a two-parameter model that includes the yield stress and plastic viscosity. This study also estimated the inter-particle distances of the suspensions by measuring the wet packing density of the cementitious suspensions. Moreover, the relationship between the inter-particle distances and the rheological properties was established.

## 2. Materials and Methods

### 2.1. Materials and Sample Preparations

**Figure 1:**
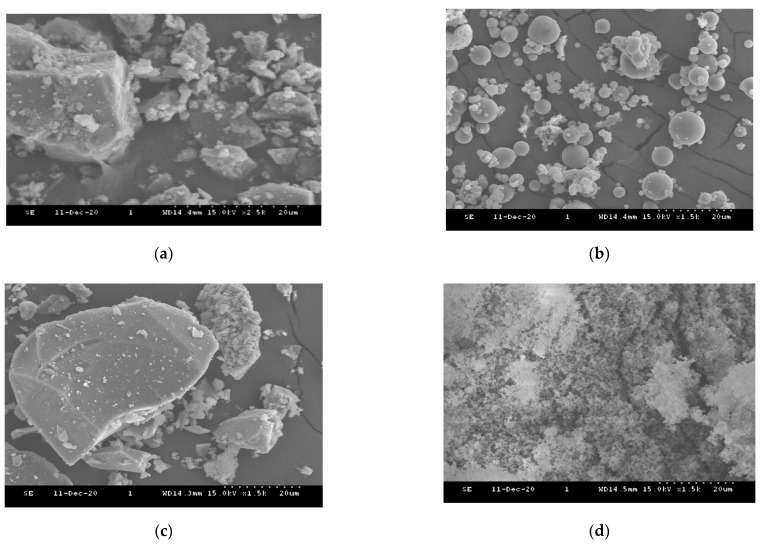
SEM micrographs of cementitious materials: (**a**) PC; (**b**) FA; (**c**) BS; (**d**) SF.

**Figure 2:**
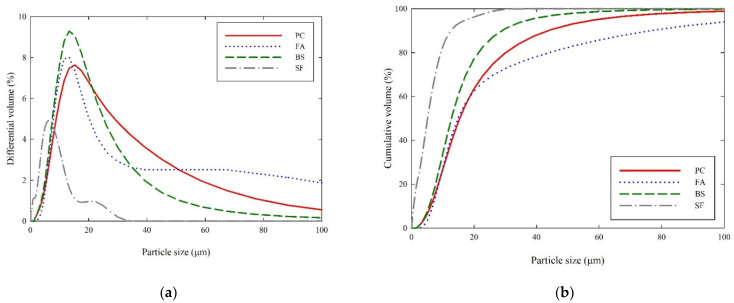
Particle size distribution of cementitious materials: (**a**) differential volume (%); (**b**) cumulative volume (%).

Type I ordinary Portland cement (PC), fly ash (FA), blast furnace slag (BS), and silica fume (SF) were used as cementitious materials for the experiments. Figure 1 shows the SEM micrographs of cementitious materials. In the SEM micrographs, PC and BS were mostly angular, FA was spherical, and SF showed an agglomeration of spheroid particles. Their chemical compositions were measured by X-ray fluorescence spectroscopy (model, PW2400, Philips Analytical, WR14 1XZ, UK) and are shown in Table 1. The particle size distribution and their mean sizes were measured using laser scattering particle size distribution analyzer (model, LS 13 320, Beckman Coulter, CA, USA), plotted in Figure 2. The physical properties including density, specific surface area (SSA), and mean particle sizes are shown in Table 2. This study was designed to determine the rheological properties of cementitious materials with different W/S ratios. All the cementitious suspensions were designed as non-blended mixes (i.e., water + PC, water + FA, water + BS, and water + SF) and the water-to-solid (W/S) ratios were quantified in terms of volumetric ratios, as the rheological properties were governed by the volume ratio rather than weight ratios. Therefore, in this study, the W/S ratios varied from 1.25, 1.50, 1.75, 2.00, 2.25, to 2.50, respectively. The considered water-to-solid volume ratios and their corresponding weight ratios (w/b ratio) are mentioned in Table 3. A total of 24 samples were prepared as 6 samples for each cementitious material. Prior to mixing, all materials were preconditioned at constant temperature (20 ± 3 °C) for 24 h in the laboratory to minimize the temperature difference. The cementitious mixtures were prepared using a Hobart mixer. Firstly, the cementitious material and 70% of the mixing water were mixed for 3 min. Then, the remaining 30% water was added to the mixtures and mixed well for 2 min. The mixer was stopped, and the sides of the mixing bowl were scraped using a paddle. After that, the mixtures were completely mixed until they became flowable.

### 2.2. Rheological Tests

Brookfield DV2T rheometer (model, Brookfield DV2T, cBrookfield Engineering Laboratories, Inc., Middleboro, MA, USA) was used to measure the shear stress response to applied strain rate. The data collection and control were performed with the PC connected to the rheometer. After the mixing process, the test mixture was poured into a cylindrical container and the spindle was introduced into the container for the measurements. This study adopted the Bingham model, a two-parameter model that includes yield stress and plastic viscosity. There was a linear relationship between shear stress and shear rate in the Bingham model as shown in Equation (1), as follows:

(1) $$\tau =\tau_{0}+\mu \gamma$$

where $\tau_{0}$ and μ indicate the yield stress and plastic viscosity of the cementitious suspensions. The torque required to rotate the spindle in the cementitious suspensions, and the two shear rate curves with increasing and decreasing rate, were determined. The shear rate was ramped up from 0 to 33.15 s^−1^, and then ramped down from 33.15 to 0 s^−1^, respectively. Three repeated tests were performed with each sample. The curves at the decreasing rate were more consistent and were used to evaluate the rheological properties. Linear regression was performed to determine the plastic viscosity and yield stress with the slope and intercept of the regression analysis line plotted through shear stress against shear rate.

### 2.3. Wet Packing Density Tests

The wet packing method was used to measure the wet packing density ($\rho_{p}$) of the prepared cementitious suspensions [32,41]. The prepared mixtures were transferred to a cylindrical mold and the mold was filled to excess. The compaction was applied using a vibrator for 60 s to ensure the exhaust of air voids. After that, the excess paste was removed with a straight edge and the amount of paste in the mold was weighted. Three repeated tests were carried out for each sample and the wet packing densities of the cementitious suspensions were calculated by Equation (2), as follows:

(2) $$\rho_{p}=\frac{W_{t}-W_{c}}{V_{c}}$$

where $V_{c}$ represents the volume of container; $W_{t}$ indicates the total weight of container and suspensions; $W_{c}$ represents the weight of container. From the test results, the void ratio, solid volume concentration, and inter-particle distances were determined.

## 3. Estimation of Inter-Particle Distances (*H*)

There are various studies that have developed an analytical method for the estimation of the distance between the particles in cementitious suspensions [41,42]. When considering the dry packing of the particles, it was assumed that the particles in the water were in contact with other particles separated by the water surrounding the particles. By measuring the thickness of the water film surrounding the particles, the inter-particle distances (H) were obtained by calculating twice the thickness of the water film thickness ($T_{w}$), as depicted in Equation (3), as follows:

(3) $$H=2\times T_{w}$$

The water film thickness ($T_{w}$) of the suspensions was determined using the wet packing density, using the following steps: Firstly, the solid volume concentration of the suspensions was determined using the wet packing density. These suspensions consist of cementitious materials and water, whose solid volume concentration (ϕ) was calculated as shown in Equation (4), as follows:

(4) $$\phi =\frac{\rho_{p}-\rho_{w}}{\rho_{c}-\rho_{w}}$$

where $\rho_{p}$ denotes the wet packing density of the suspensions; $\rho_{c}$ and $\rho_{w}$ denote the densities of the cementitious particles and water. From the maximum solid volume concentration ($\phi_{m}$), the corresponding minimum void ratio ($Void_{min}$) was determined using Equation (5). The minimum void ratio is defined as the ratio of the minimum void volume to the solid volume of the particles. From the minimum void ratio, the volume of excess water was determined using Equation (6). The volume of excess water ($V_{ew}$) is defined as the ratio of the volume of excess water to the solid volume of the cementitious materials.

(5) $$Void_{min}=\frac{1-\phi_{m}}{\phi_{m}}$$

(6) $$V_{ew}=V_{w}-Void_{m}$$

where, $\phi_{m}$ represents the maximum solid volume concentration, and $V_{w}$ indicates the volume ratio of water-to-solid particles. By determining the volume of excess water, the water film thickness surrounding the particles was determined using Equation (7). The water film thickness has the physical meaning of the average thickness of the water films coating the solid particles.

(7) $$T_{w}=\frac{\;V_{ew}}{SSA}$$

where SSA represents the specific surface area of the cement particles in a unit volume of suspensions.

## 4. Results and Discussions

### 4.1. Rheological Properties of Cementitious Suspensions with Different W/S Ratios

**Figure 3:**
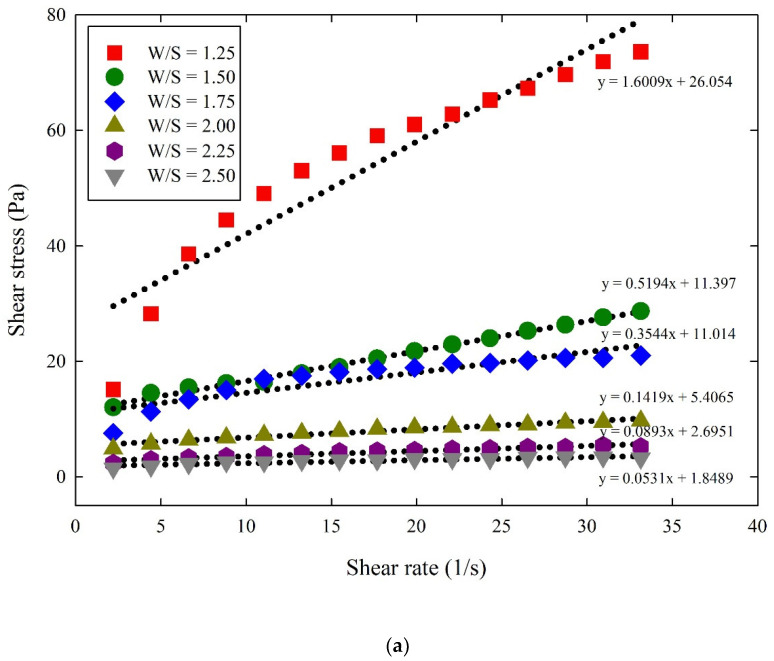

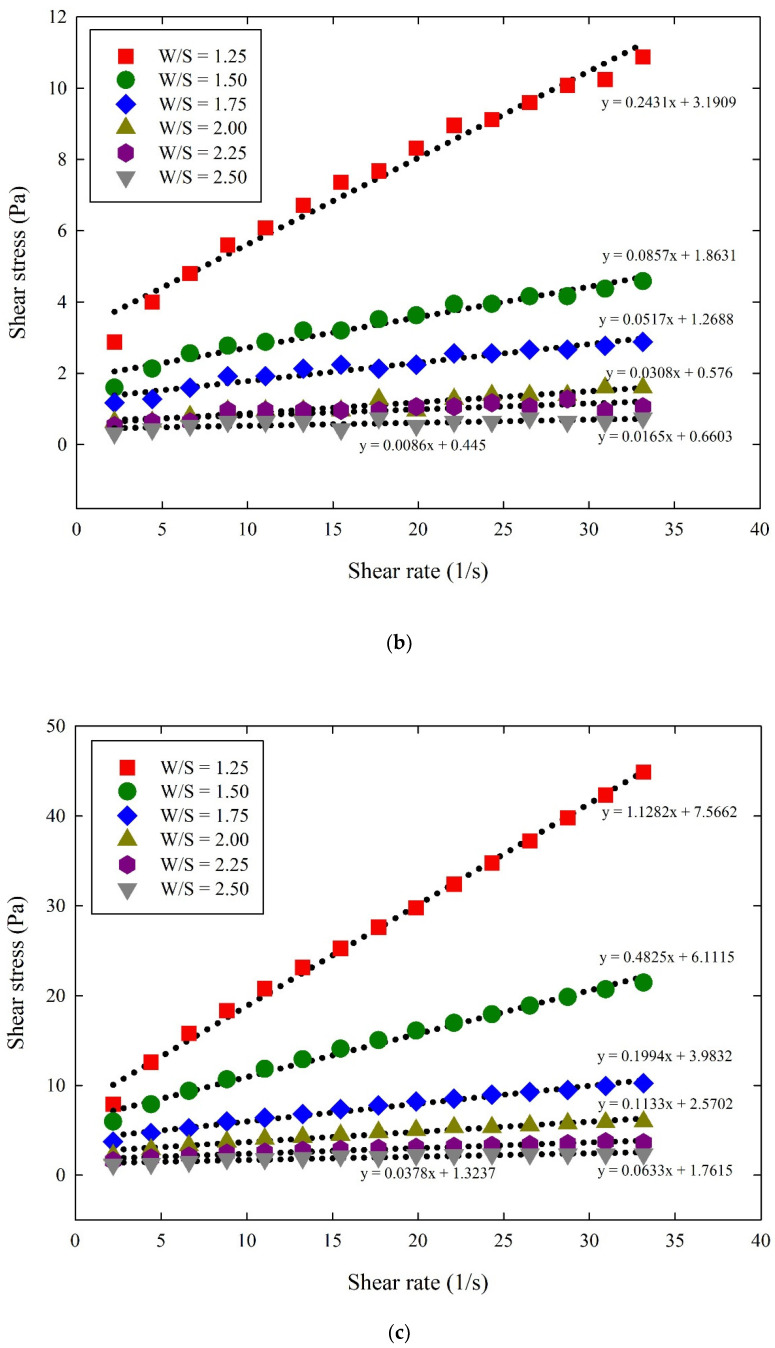

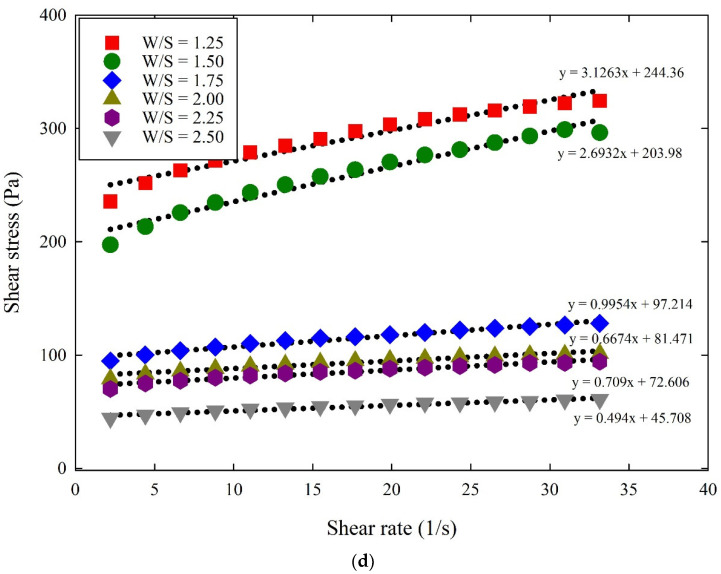
Effect of W/S ratios on the shear stress of cementitious suspensions: (**a**) PC; (**b**) FA; (**c**) BS; (**d**) SF.

Figure 3 shows the effect of the W/S ratios on the shear stress of the cementitious suspensions. The figure shows that an increase in shear rate increases the shear stress, and the maximum shear stress occurs at the maximum shear rate. For all the cementitious suspensions, the shear stress at the maximum shear rate decreases significantly as the W/S ratio increases. Subsequently, a linear regression analysis was carried out to determine the yield stress and plastic viscosity using the slope and intercept of the regression plotted through shear stress against shear rate.

**Figure 4:**
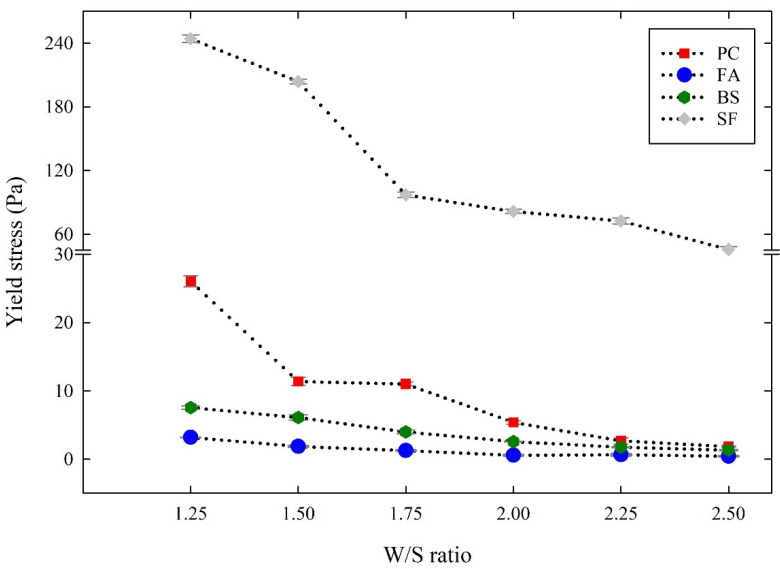
Yield stress of cementitious suspensions with different W/S ratios.

**Figure 5:**
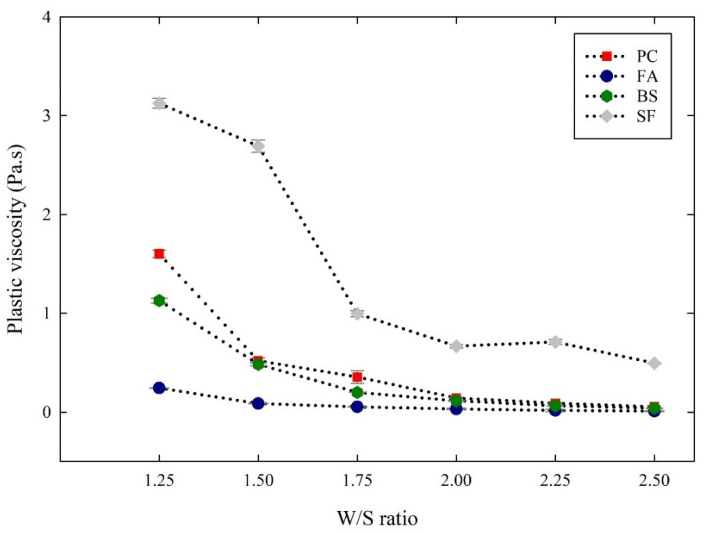
Plastic viscosity of cementitious suspensions with different W/S ratios.

Figure 4 and Figure 5 show the obtained yield stress and plastic viscosity of cementitious suspensions with different W/S ratios. All the cementitious suspensions tended to decrease the yield stress and plastic viscosity, showing improved flowability as the W/S ratio increased. At the same time, the yield stress and plastic viscosity obtained at the same W/S ratios were different for each cementitious suspension. As a result of comparing the FA suspensions with the PC suspensions, the yield stress of the FA suspensions decreased by 75~90% and the plastic viscosity decreased by 78~86% at the same W/S ratio as the PC suspensions. The mechanisms governing the enhanced fluidity of the FA suspensions were grain morphology, pozzolanic activity, and filling effects [20,21,22,23,24,25]. Moreover, although the particle sizes of FA and PC were almost the same, the shape of FA was mostly spherical, which facilitates the separation and dispersion of particles, thus providing a more liquid mixture at the same W/S ratio [20,21,22,23,24,25]. These factors influence the improved flowability of the FA suspensions compared to the PC suspensions. Similarly to the FA suspensions, the BS suspensions also provide lower yield stress and plastic viscosity than the PC suspensions. The yield stress and plastic viscosity of the BS suspensions decreased by 28~70% and 8~44%, respectively, as compared to the PC suspensions. This is associated with the smooth surface of the BS particles and their lower chemical activity than that of PC [8,9,26]. In addition, the micro-filling effect and the surface area also influence the rheological properties of the BS suspensions [8,9,26,27,28]. On the contrary, the FA and BS suspensions showed a decreasing tendency, while the SF suspensions provided a significant increase in the yield stress and plastic viscosity compared to the PC suspensions. The yield stress of the SF suspensions increased significantly in the range of 782~2372%, and the plastic viscosity increased in the range of 95~830%. As the SF consists of finer particles, the SF particles can fill the voids without loosening the packing of particles, which ultimately increases the yield stress and plastic viscosity of the suspensions [28,29]. During shearing, the formation of silica sol by ultra-fine silicon particles enhances the thixotropy of the paste, which was unfavorable to the initial flow of the paste, thereby increasing the rheological properties [30,31,32].

### 4.2. Inter-Particle Distances (H) of the Cementitious Suspensions

**Figure 6:**
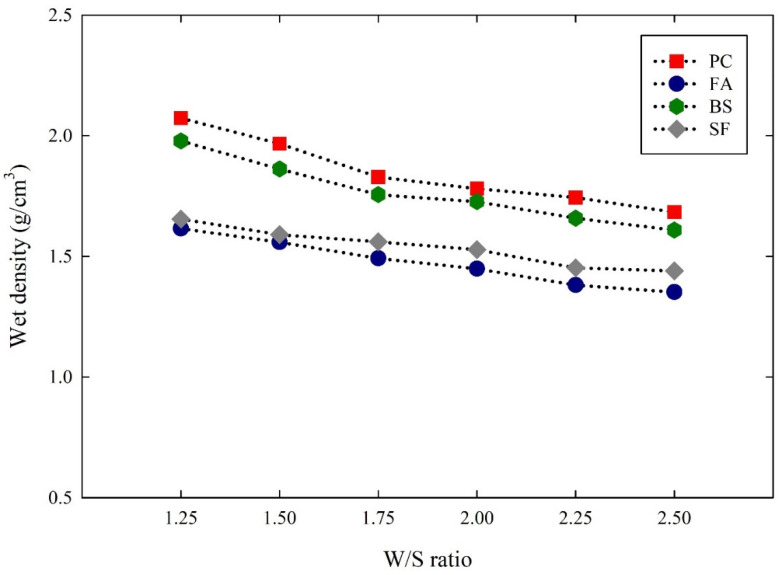
Wet density of cementitious suspensions with different W/S ratios.

**Figure 7:**
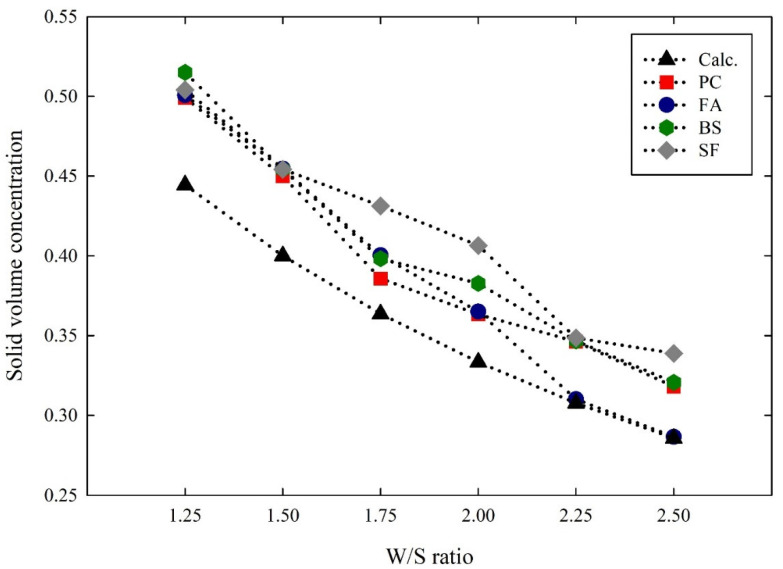
Solid volume concentration of cementitious suspensions with different W/S ratios.

Since the rheological properties of the suspensions were influenced by the particle characteristics, the inter-particle distance plays a major role in the rheological properties that consider the particle size and surface area of the particles. By estimating the inter-particle distances of the suspensions, their relationship with the rheological properties can be computed. The inter-particle distances of the cementitious suspensions were estimated using an analytical method developed by various studies [41,42]. To determine the inter-particle distance of the suspensions, the wet density of the suspensions was measured. Figure 6 shows the wet density of the cementitious suspensions with different W/S ratios. The wet density of all the suspensions decreased in the range of 1 to 7% as the W/S ratios increased. By comparing the wet density between the suspensions, the PC suspensions obtained the highest wet density, followed by the BS, SF, and FA suspensions, which related to the density of materials. Then, the solid volume concentrations were calculated using the wet density of the suspensions. Figure 7 shows the solid volume concentration of cementitious suspensions with different W/S ratios. Theoretically, the solid volume concentration at each of the W/S ratios was calculated to be 0.44, 0.40, 0.36, 0.33, 0.30, and 0.28, respectively. The solid volume concentration of the PC suspensions increased by an average of 10% from the calculated values. Similarly, the solid volume concentrations of the FA, BS, and SF suspensions increased by an average of 8%, 13%, and 16%, respectively, from the calculated values.

**Figure 8:**
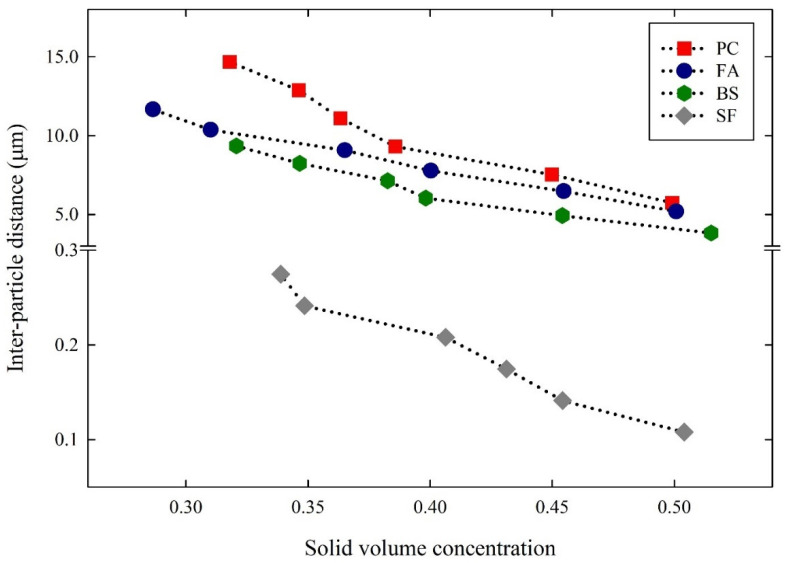
Inter-particle distance to solid volume concentration of cementitious suspensions.

**Figure 9:**
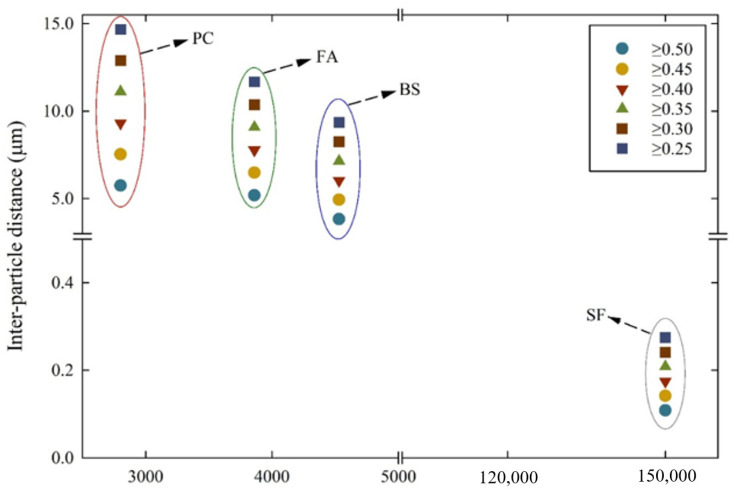
Inter-particle distance to specific surface area of cementitious suspensions.

From the solid volume concentrations obtained above, the inter-particle distances of the suspensions were calculated and plotted in Figure 8. The inter-particle distance of the PC suspensions increased as the solid volume concentration decreased, corresponding to 5.74~14.67 µm. In the FA suspensions, the inter-particle distance was 5.18~11.66 µm. In the case of the BS and SF suspensions, the inter-particle distances were 3.82~9.34 µm and 0.107~0.27 µm, respectively, according to the decrease in solid volume concentration. For all the cementitious suspensions, the inter-particle distances increased with a decrease in the solid volume concentration. The inter-particle distances obtained from the PC and FA suspensions were almost the same, while the BS and SF suspensions obtained relatively smaller inter-particle distances. These were related to the particle size and SSA of the cementitious particles. The particle sizes of PC, FA, BS, and SF were 21.58 µm, 30.2 µm, 15.85 µm, and 6.302 µm, respectively. At the same solid volume concentration, the inter-particle distances dropped significantly with a decrease in particle sizes, due to the larger SSA of the cementitious materials. The relationship between SSA and the inter-particle distances is shown in Figure 9. The SSA of PC was 2800 cm^2^/g, and the inter-particle distance lies between 5.74 and 14.67 µm. As the SSA of FA was 3860 cm^2^/g, the inter-particle distance was slightly reduced in the range of 5.18~11.66 µm. In the case of BS, the SSA was 4530 cm^2^/g, and the inter-particle distance was reduced to 3.82~9.34 µm. In addition, the SSA of SF was 150,000 cm^2^/g, and the inter-particle distance was reduced significantly in the range of 0.107~0.27 µm. The higher the SSA, the lower the inter-particle distances, which significantly alters the suspension rheology.

### 4.3. Relationship between Inter-Particle Distances to the Rheological Properties

**Figure 10:**
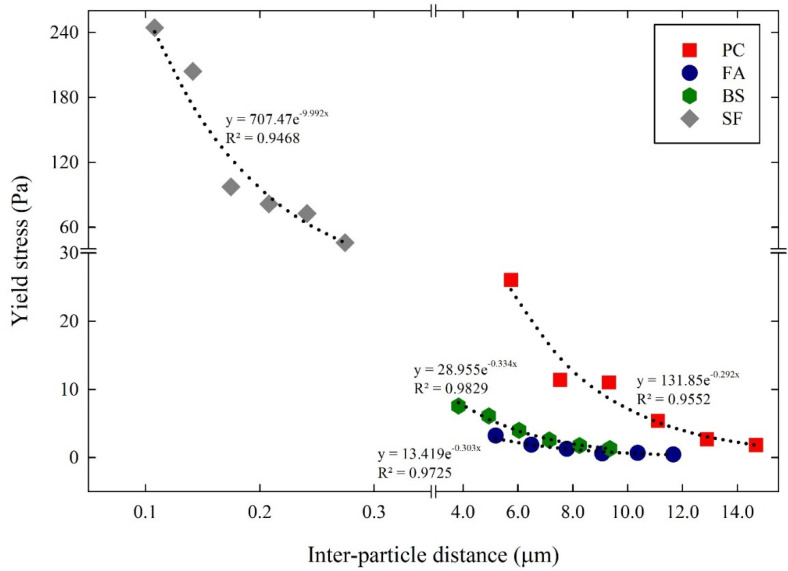
Relationship between inter–particle distance and yield stress of cementitious suspensions.

The relationship between the inter-particle distance and the yield stress of the cementitious suspensions is illustrated in Figure 10. The maximum yield stress in the PC suspensions was obtained at a distance of 5.74 µm. Similarly, the maximum yield stress of the FA, BS, and SF suspensions was obtained at distances of 5.18 µm, 3.82 µm, and 0.107 µm, respectively. The relationship between yield stress and inter-particle distance was introduced by [32], with an exponential function. Using the exponential function, the ultimate yield stress when the inter-particle distance reached zero, and the sensitivity of yield stress to the variations in inter-particle distance, were obtained as shown in Equation (8), as follows:

(8) $$\tau =ae^{-b.H}$$

where H represents the inter-particle distance, a represents the ultimate yield stress when the H of the cementitious suspensions reached zero, and b indicates the sensitivity of yield stress to the change in H. The ultimate yield stress (a) and the sensitivity to yield stress (b) of the cementitious suspensions are listed in Table 4. The ultimate yield stress obtained for the PC suspensions was 131.85 Pa, and the sensitivity to yield stress was 0.292 µm^−1^. In the FA, BS, and SF suspensions, the ultimate yield stress was 13.419 Pa, 28.955 Pa, and 707.47 Pa, and the sensitivity to yield stress was 0.303 µm^−1^, 0.334 µm^−1^, and 9.992 µm^−1^, respectively. The sensitivity to yield stress obtained for the SF suspensions was higher when the suspensions had a smaller particle size and a large SSA. In contrast, the sensitivity to yield stress obtained for the PC suspensions was relatively low, due to the larger particle size and low SSA. This indicates that the yield stress of the cementitious suspensions was more sensitive to fine particles. Those fine particles with a large SSA of the cementitious suspensions improved particle packing, increasing the sensitivity to yield stress.

**Figure 11:**
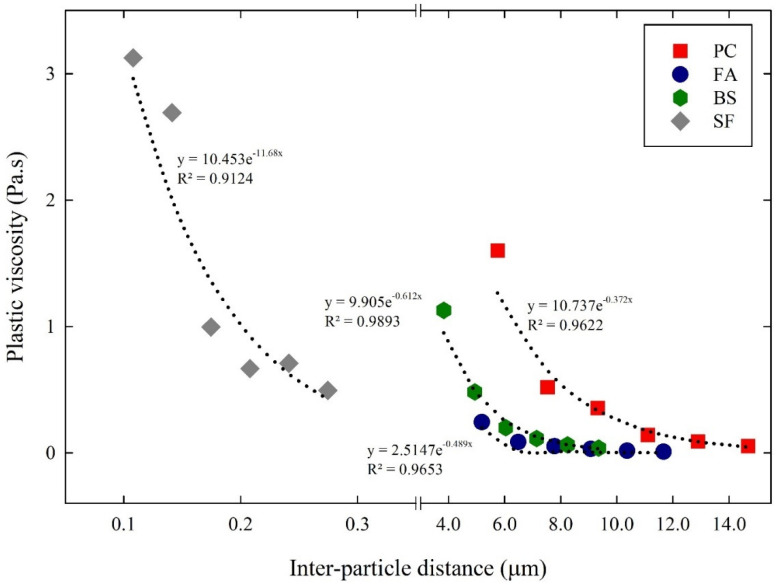
Relationship between inter–particle distance and plastic viscosity of cementitious suspensions.

A similar empirical approach was also employed to define the relationship between the inter-particle distances and the plastic viscosity of the cementitious suspensions. Figure 11 shows the relationship between the inter-particle distance and plastic viscosity of the cementitious suspensions. The relationship between plastic viscosity and inter-particle distance was investigated using the exponential function, as shown in Equation (9), as follows:

(9) $$\mu =ae^{-b.H}$$

where a represents the ultimate plastic viscosity as H reaches zero and b represents the sensitivity of plastic viscosity to the variation in H. The ultimate plastic viscosity (a), and the sensitivity of plastic viscosity (b), are listed in Table 5. The ultimate plastic viscosity obtained for the PC suspensions was 10.73 Pa.s, and the sensitivity was 0.372 µm^−1^. In the cases of the FA, BS, and SF suspensions, the ultimate plastic viscosity obtained was 2.51 Pa.s, 9.905 Pa.s, and 10.45 Pa.s, respectively. The sensitivity of plastic viscosity obtained was 0.489 µm^−1^, 0.612 µm^−1^, and 11.68 µm^−1^, respectively. As a result of the above, the SF suspensions had higher sensitivity and relatively lower sensitivity than the PC suspensions. This also indicates that the plastic viscosity of the cementitious suspensions was more sensitive to the fine particles with a large surface area, improving particle packing.

## 5. Conclusions

This study was designed to determine the rheological properties of PC, FA, BS, and SF suspensions with different W/S ratios, which varied from 1.25, 1.50, 1.75, 2.00, 2.25, to 2.50. The W/S ratios were more significant in decreasing the rheological properties of all the cementitious suspensions. All the cementitious suspensions tended to improve flowability as the W/S ratio increased. Compared to the PC suspensions, the rheological properties of the FA and BS suspensions showed improved flowability. The shape of FA was spherical, which could serve for isolation and dispersion, thus providing more liquid mixtures. The smooth surface of the BS particles, less chemical activity, micro-filling effect, and large surface area could improve the flowability of the BS suspensions. On the contrary to the FA and BS suspensions, the SF suspensions caused a significant increase in the yield stress and plastic viscosity as compared to the PC suspensions. As the SF consists of more fine particles, it could fill the voids without loosening the packing of particles, which increased the yield stress and plastic viscosity compared to the PC suspensions. In addition to these factors, the inter-particle distances of the suspensions also influence the rheological properties that consider the particle size and SSA of particles. For all the cementitious suspensions, the inter-particle distances increased linearly with a decrease in the solid volume concentration. The inter-particle distances of the PC and FA suspensions were almost the same, whereas the BS and SF suspensions obtained low inter-particle distances. The inter-particle distances were related to the size and SSA of the particles. With the same solid volume concentration, the inter-particle distance dropped significantly with the decrease in particle size, which was also attributed to the increase in SSA of the cementitious materials. The relationship between the inter-particle distance and rheological properties of cementitious suspensions was illustrated using an exponential function. The sensitivity of the yield stress and plastic viscosity obtained for the SF suspensions was high, and that of the PC suspensions was relatively low. This indicates that the rheological properties of the cementitious suspensions were more sensitive to fine particles with a large surface area.

With the help of completely understanding the rheological properties of the individual SCMs, it would be possible to control the mixture of SCMs according to the required flowability or rheological properties. The results of this study can be expanded to further works on the scientific quantification of rheological properties for various kinds of mixtures with SCMs.

## Figures and Tables

**Table 1:** Chemical compositions of cementitious materials.

Composition	PC (%)	FA (%)	BS (%)	SF (%)
SiO_2_	20.50	53.70	33.80	99.10
Al_2_O_3_	5.11	25.70	13.90	-
CaO	62.00	5.76	44.20	-
MgO	2.60	-	3.57	-
Fe_2_O_3_	3.30	-	-	-

**Table 2:** Physical properties of cementitious materials.

	PC	FA	BS	SF
Density (g/cm^3^)	3.15	2.23	2.90	2.30
SSA (cm^2^/g)	2800	3860	4530	150,000
Mean size (µm)	21.58	30.20	15.85	6.31

**Table 3:** Mix ratios of cementitious suspensions.

Water-to-Solid Volume Ratios	Weight Ratios (w/b)			
	PC	FA	BS	SF
1.25	0.39	0.56	0.43	0.54
1.50	0.47	0.67	0.51	0.65
1.75	0.55	0.78	0.60	0.76
2.00	0.63	0.89	0.68	0.86
2.25	0.71	1.00	0.77	0.97
2.50	0.79	1.12	0.86	1.08

**Table 4:** Exponential parameters of cementitious suspensions (yield stress).

Suspensions	*a* (Pa)	*b* (µm^−1^)
PC	138.85	0.292
FA	13.419	0.303
BS	28.955	0.334
SF	707.47	9.992

**Table 5:** Exponential parameters of cementitious suspensions (plastic viscosity).

Suspensions	*a* (Pa.s)	*b* (µm^−1^)
PC	10.737	0.372
FA	2.5147	0.489
BS	9.905	0.612
SF	10.453	11.68

## Data Availability

Not applicable.
